# Association between Childhood Suicidal Ideation and Geriatric Depression in Japan: A Population-Based Cross-Sectional Study

**DOI:** 10.3390/ijerph17072257

**Published:** 2020-03-27

**Authors:** Ayako Morita, Takeo Fujiwara

**Affiliations:** Department of Global Health Promotion, Tokyo Medical and Dental University, Yushima-ku, Tokyo 113-8510, Japan

**Keywords:** late life, mental health, children and adolescents, Japan

## Abstract

Adverse childhood experiences (ACEs) are assumed to increase the risk of depression in late life via development of poor mental health conditions; however, the association between mental distress in childhood and geriatric depression has not been directly examined. This study examined the association between childhood suicidal ideation and geriatric depression, using population-based, cross-sectional survey data from 1140 community-dwelling, functionally independent older adults in Wakuya City, Japan. We assessed childhood suicidal ideation by asking the participants whether they had seriously considered attempting suicide before the age of 18, together with geriatric depression, using the Japanese version of the 15-item Geriatric Depression Scale. Poisson regression was applied to adjust for potential confounders and mediators. In total, 6.1% of the participants reported childhood suicidal ideation. After adjustment for sex, age, personality attributes and ACEs, childhood suicidal ideation was positively associated with geriatric depression prevalence ratio [PR]: 1.40, 95% Confidence Interval (95%CI): 1.04–1.88). The increased PR of geriatric depression remained significant, even after further adjustment for adulthood socio-economic status, recent life stressors and current health status (PR: 1.38, 95%CI: 1.02–1.88). Further prospective studies are warranted, but efforts to deliver mental health services to children with suicidal ideation potentially diminish the highly prevalent geriatric depression.

## 1. Introduction

Geriatric depression is one of the most common psychiatric disorders among older adults. The prevalence of clinically relevant depressive symptom cases among community-dwelling older adults was reported to be 7.2–49.0% [1], and it was generally higher among women and in deprived areas [2]. Geriatric depression leads to further burdens on older adults and society, including lower quality of life, reduced functional ability in daily life [3], increased medical service use [4] and higher mortality rates [5]. Globally, populations are rapidly aging, and many societies are facing significant challenges due to staggering increases in healthcare and long-term care costs for older adults. There is an urgent need to develop interventions that prevent geriatric depression, not only for the current generation, but also for future generations.

Recent studies applying a life course framework demonstrate that adverse childhood experiences (ACEs), such as loss of a parent and divorce, maltreatment in the home, and other family dysfunctions and social deprivations, predict future geriatric depression [6,7,8,9]. Exposure to ACEs may increase the risk of experiencing depression in late life along several causal pathways. These include high physiological and cognitive stress sensitivity due to altered gene expression; altered brain structure and function, associated with stress regulation areas such as the hypothalamic–pituitary–adrenal (HPA) axis, medial prefrontal cortex and amygdala [10,11]; and severe chronic stress exposure both externally [12] and endogenously [13], which could lead to unhealthy lifestyles/maladaptive stress coping behaviors and deteriorated physical and cognitive functions. The empirical evidence is, however, limited in terms of the role of early-onset poor mental health, which may function as an endogenous psychosocial stressor in the link between ACEs and geriatric depression.

A prior episode of depression is a well-known significant risk factor for geriatric depression [14]. Earlier research has shown that ACEs are predictive of poor mental health conditions in childhood [15,16], and children with ACEs are at increased risk of recurrent or persistent depressive episodes [17]. Moreover, our capacity for voluntary memory suppression is known to decrease with age [18], suggesting that the influence of endogenous psychosocial stressors rooted in early life could continue or even be enhanced in late life. Earlier studies have shown that mental disorders with onset before the age of 20 years are associated with the adulthood incidence of numerous chronic physical illnesses, independent of ACEs [13]. However, there remains a lack of research that has directly investigated the relationship between poor childhood mental health and depression in late life, which could provide valuable information for developing interventions to prevent or mitigate the long-term adverse effects of ACEs on mental health.

Although prospective study is ideal, it is highly time-consuming and costly to follow from childhood to late life. Autobiographical memory is largely preserved across the lifespan, especially of experiences that occurred during adolescence [19] and were associated with strong emotion at the time of memory formation [20]. Suicidality is one of the highly recallable experiences [21] and is closely related to experience of depressive symptoms, or other psychological disturbances [22,23]. Recall of childhood suicidal ideation, therefore, might be a useful indicator of childhood mental health.

We hypothesized that childhood suicidal ideation is associated with an increased risk of geriatric depression and aimed to investigate the hypothesis among a population of community-dwelling older adults in Japan.

## 2. Materials and Methods

### 2.1. Data and Study Participants

In 2017, we distributed a self-administered questionnaire to all the residents of Wakuya City (Miyagi prefecture, Japan) aged 65 years and above who were enrolled in the National Health Insurance Plan (*n* = 4839) and collected the responses by mail. In total, 1722 (35.6%) agreed to participate and returned the filled questionnaires. To minimize recall biases, the present study excluded those who were receiving medical treatment for dementia (*n* = 48), living in an institution or receiving long-term care assistance at home (*n* = 179), and those having missing responses for the main measures (*n* = 355). The final dataset included the responses of 1140 older adults. The study was approved by the research ethics committee at Tokyo Medical and Dental University. The ethical approved project identification code is M2000-2077.

### 2.2. Measures

#### 2.2.1. Suicidal Ideation in Childhood (Independent Variable)

We assessed suicidal ideation in childhood as s proxy of mental health state in childhood. Following the Japan Youth Risk Behavior Survey [24], we asked the participants: “have you seriously considered attempting suicide before the age of 18?” with a dichotomous response category (yes/no).

#### 2.2.2. Geriatric Depression (Dependent Variable)

Geriatric depression status was assessed using the Japanese Version of the 15-item Geriatric Depression Scale (GDS-15), a clinically applicable screening tool for depression among older adults with an optimal cutoff of 6/7 [25]. Participants were asked to answer 15 questions with a dichotomous response category (yes/no), and those who scored seven or more were classified as clinically depressive.

#### 2.2.3. Covariates

In addition to the main measures above, we measured sex, age, personality and childhood adversities as the potential confounders of the association between childhood suicidal ideation and geriatric depression. We employed the Japanese version of the Ten-Item Personality Inventory (TIPI-J) [26] which has shown good concurrent validity, internal consistency and test-retest reliability among older adults [27], to compute a score (1–7) for each of the Big Five personality dimensions (i.e., neuroticism, extraversion, openness to experience, agreeableness and conscientiousness) and to classify them into four quartiles (lowest, second, third and highest). With respect to ACEs, we measured eight dimensions following the questions adapted in earlier studies: maltreatment experiences at home before the age of 18 (i.e., parental death, parental divorce, mentally ill parents, witness of intimate partner violence, physical abuse, neglect and psychological abuse, economic difficulty) [28]. We then classified the participants into no ACE, 1 ACE and 2+ ACEs to investigate cumulative effects, as all the dimensions were equally strongly associated with geriatric depression (Supplementary Table S1) and 2+ ACEs are reported to increase risk of mental illness in young adulthood [10].

We further measured adulthood socio-economic status (SES) and current health and social relationships as potential mediators in the pathway from poor childhood mental status, indicated by suicidal ideation, to geriatric depression. Adulthood SES was measured by asking the participants about years of formal education (9 years or less, 10–12 years, 13 years+, or other); types of longest-held occupation, which were classified into manual, industrial or skilled labor [8]; annual household income, which was classified into less than 2.00 million yen, 2.00–4.99 million yen, 5.00–8.99 million yen, 9.00 million yen or higher; incidence of 13 common stressful life events in late life in the past 12 months, which were rated according to the social readjustment rating scale for the Japanese elderly [29]; and current health status based on a checklist of 21 medical conditions [8]. Finally, current social relationships were measured by asking the participants about marital status (married, divorced/separated/single/other); household composition (living alone, living with others), employment status (yes/no); social support provision (yes/no); social support receipt (yes/no) and help-seeking attitudes, according to the degree of agreement with a question: “Do you feel shameful consulting or seeking help with your worries and fears?” (strongly disagree/disagree = active, neither = neutral, agree/strongly agree = passive).

### 2.3. Statistical Analysis

First, we compared the characteristics of the participants who had a history of suicidal ideation in childhood with those who did not, using the t-test for continuous independent variables and chi-square test for categorical independent variables. Second, we compared geriatric depression prevalence by history of suicidal ideation in childhood using the chi-square test and Poisson regression with robust error variance analysis to compute prevalence ratio (PR), adjusted for sex, age and personality, in Model I, Model I variables + ACEs in Model II, Model II variables + adulthood SES in Model III, and Model III variables + current health status and social relationships in Model IV. Poisson regression was chosen, because use of odds ratios to approximate risk ratios and associated confidential intervals can lead to overestimating of the effect of the independent variable when the main outcome is highly prevalent (>10%) [30].To handle missing data of some covariates (i.e., TIPI-J score, education, occupation type, household income, recent life stress, marital status, employment status, provision and receipt of emotional support, help-seeking attitude), we conducted multiple imputation. We replicated the incomplete dataset 100 times, with the missing values replaced by values drawn from the complete data (i.e., age, residential area, GDS score, ACE score, sex, total number of medication conditions, cardiac disease status, household composition) (100 imputed datasets), performed Poisson regression analysis on each of the imputed datasets and reported the combined parameter estimates. STATA 14.0 (StataCorp LLC, College Station, TX, USA) was used to perform all the analyses.

## 3. Results

Table 1 presents the overall characteristics of the participants. In total, 69 (6.1%) of the participants reported a history of suicidal ideation in childhood, and geriatric prevalence among them was 11.6 points higher than the participants without a history of suicidal ideation in childhood. There was no significant difference in age, sex and Big Five personality attributes between the groups; however, older adults with a history of suicidal ideation in childhood were more likely to have experienced all kinds of ACEs except parental death, in comparison with older adults without a history of suicidal ideation. With respect to adulthood adversities, older adults with a history of suicidal ideation in childhood were more likely to suffer from heart disease (21.3%) and multiple types of medical conditions (on average: 1.96), but there was no significant difference in adulthood SES and current social relationships.

Prevalence of geriatric depression among those with childhood suicidal ideation was higher than among those without childhood suicidal ideation (39.1% vs. 24.8%, Cohen’s d = 0.33 (95%CI: 0.08, 0.57)). Table 2 presents the results of Poisson regression model analysis.

Older adults with a history of childhood suicidal ideation displayed a statistically significantly higher PR of geriatric depression when adjusted for demographics and personality attributes (adjusted PR (APR): 1.55, 95%CI: 1.17–2.07). They continued to show a greater risk of geriatric depression even after adjustment for childhood adversities (APR: 1.40, 95%CI: 1.04–1.88). Model III resulted in decreased yet significantly higher prevalence of geriatric depression among older adults who had a history of suicidal ideation as a child compared with those who did not (APR: 1.33, 95%,CI: 1.03–1.86). Model IV resulted in a slight increase of PR to 1.38 (1.02–1.88). PRs with 95%CI for association between covariates and geriatric depression are summarized in Supplementary Table S2.

## 4. Discussion

In this population-based study, poor childhood mental health indicated by childhood suicidal ideation was independently associated with an increased risk of geriatric depression. The attenuated yet remaining significant positive association between childhood suicidal ideation and geriatric depression after adjustment for ACEs suggests that the development of poor mental health in childhood is one of the underlying mechanisms that links ACEs and geriatric depression.

Our findings are consistent with a prospective cohort study that followed the community-dwelling participants from the age 15 to 30. The study showed that suicidal ideation at age 15 was associated with emotional and behavioral deficits in late adolescence [23] and increased risk of mood disorders in young adulthood [31]. While suicidal ideation is a risk factor for premature deaths from suicide and other medical conditions [32], our study participants survived to old age. Our findings contribute to the existing literature with new evidence that children who experienced suicidal ideation are at increased risk of depression, even when they survived to old age, attaining comparative adulthood SES and social relationships to those who did not experience suicidal ideation in childhood and retaining physical and cognitive functional independence.

The significant association of poor childhood mental health with geriatric depression, independent of ACEs, is consistent with earlier studies [13,33]. A multi-national, cross-sectional community study [13] showed that structured diagnostic interview-based detection of early-onset (before the age of 20) mental disorders, including major depressive disorder, generalized anxiety disorder, social phobia, posttraumatic stress disorder, panic disorder and agoraphobia, was associated with an increased hazard ratio of chronic spinal pain and frequent or severe headache, which are common physical symptoms expressed by depressive people. Additionally, a large national study from Scotland also showed that “fair” or “poor” general subjective health status in childhood, which could partially reflect one’s recollection of general mental health in childhood, is a significant independent risk factor for late-life depressive symptoms [34]. The little change in estimated PR from Model III to Model IV, after adjustment for adulthood SES, current health and social relations, suggests that poor mental health in childhood has a strong and direct effect on geriatric depression, possibly due to experience of difficulties with negative emotional regulation, which becomes more problematic in late life, when people decline in their abilities to suppress negative memories as a result of aging [18].

To our knowledge, this is the first study that directly examined the association between childhood mental health and geriatric depression using population-based community-dwelling older adult data and found a significant positive association. However, the results of the present study should be interpreted with a consideration of its limitations. Firstly, suicidal ideation was measured by a single binary question. Although we excluded participants diagnosed with dementia from the analysis and asked about emotionally intense autobiographical experiences, we still cannot rule out the possibility of underreporting due to lack of a clear definition, feelings of shame or embarrassment or memory fades [35]. To date, no longitudinal study has provided data to verify the late-life recall of suicidal ideation in younger years. Secondly, the study was conducted in one rural municipality of Japan, and we did not achieve a high response rate. It is possible that those who suffered from depression at the survey time and those who had suffered from suicidal ideation in childhood were less likely to respond to the questionnaire, leading to potential underestimation of the true underlying associations. However, the responding participants were similar to the residents of the study site in terms of age and sex.

Thirdly, data were drawn from retrospective accounts and subject to recall bias. People may deny or reinterpret the experience over time, and their present emotional state affect the retrieval of emotional memories. We cannot rule out the possibility of ruminative retrieval of ACEs and suicidality in childhood being a hallmark rather than the cause of depression and ACEs. Depressed participants might have been more likely to recall childhood suicidal ideation, although the observed association was consistent with the findings of earlier prospective longitudinal studies that measured suicidal thoughts and behaviors in childhood/adolescence and psychosocial functioning in young adulthood [23,31]. Finally, we did not measure lifetime depressive episodes, including the first onset age, nor potential confounders such as development disabilities [36]. In future, a population-based retrospective cohort study which links childhood hospital records of suicidal ideation with late-onset depression is warranted to confirm the current findings based on a robust assessment of childhood suicidal ideation. Alternatively, qualitative research such as in-depth interviews about suicidal thoughts and mental health status across each participant’s life course could further enhance our understanding of how suicidality in childhood affects depression in late life. Poor mental health in childhood is possibly associated with other major health outcomes in later life, such as dementia, which is worth examining the in future also.

Nevertheless, Japan suffers from a high suicide rate relative to other developed countries [37]. Notably, there is an increasing suicidal rate in youth populations. A large-scale national survey among randomly selected high school students (Japan Youth Risk Behavior Survey) revealed that 16.3% of males and 27.1% of females had seriously considered suicide over the past 12 months—higher percentages than in the US [24]. Research shows that people living with mental illness often experience a specific series of changes in their thoughts, feelings and behaviors before a relapse, but more than 90% of individuals with suicidal thoughts do not receive mental health services [38]. Our findings suggest that children who have suicidal ideation need psychosocial interventions [39] to restore specific sets of feelings, emotions and behaviors and reduce the risk of poor mental health in late life.

## 5. Conclusions

The present study supports the hypothesis that poor childhood mental health exerts a direct effect on geriatric depression, which was independent of ACEs. Efforts to reduce geriatric depression should take into consideration childhood mental health. One of the targets for primary interventions could be children with suicidal ideation, to disrupt the continued endogenous stress exposure throughout life.

## Figures and Tables

**Table 1:** Characteristics of the participants by childhood mental health (*n* = 1140).

Variable				Suicidality in Childhood				
		All		No (*n* = 1071)		Yes (*n* = 69)		*p*-*Value*
		*n*	% or Mean (SD)	*n*	%	*n*	%	
**Basic demographics**								
Age (years)		1140	74.7 (7.1)	1071	74.7 (7.0)	69	74.4 (7.4)	0.67
Sex	Male	531	46.6	504	47.1	27	39.1	0.2
	Female	609	53.4	567	52.9	42	60.9	
**Personality attribute**								
Neuroticism (total no: 1–7)		1085	3.7 (1.1)	1017	3.7 (1.1)	68	3.8 (1.1)	0.36
Extraversion (total no: 1–7)		1084	4.1 (1.2)	1017	4.1 (1.2)	67	4.1 (1.0)	0.96
Openness (total no: 1–7)		1084	3.9 (1.1)	1022	3.9 (1.1)	62	3.9 (1.2)	0.91
Agreeableness (total no: 1–7)		1073	5.2 (1.0)	1006	5.2 (1.0)	67	5.4 (1.1)	0.26
Conscientiousness (total no: 1–7)		1067	4.6 (1.1)	1001	4.6 (1.1)	66	4.5 (1.3)	0.4
**Childhood adversities**								
Parental divorce (yes)		12	1.1	9	0.8	3	4.3	0.006
Parental psychopathology (yes)		10	0.9	6	0.6	4	5.8	<0.001
Parental domestic violence (yes)		47	4.1	35	3.3	12	17.4	<0.001
Physical abuse (yes)		17	1.5	14	1.3	3	4.3	0.043
Neglect (yes)		170	14.9	151	14.1	19	27.5	0.002
Emotional abuse (yes)		63	5.5	49	4.6	14	20.3	<0.001
Economic difficulties (yes)		519	45.5	469	43.8	50	72.5	<0.001
Total ACE score	0	449	39.4	440	41.1	9	13	<0.001
	1	440	38.6	413	38.6	27	39.1	
	2+	251	22	218	20.4	33	47.8	
**Adulthood Socio-Economic Status (SES)**								
Formal education	<9 years	331	29	308	28.8	23	33.3	0.73
	9–12 years	551	48.3	518	48.4	33	47.8	
	13 years+	250	21.9	237	22.1	13	18.8	
	Other/Missing	8	0.7	8	0.7	0	0	
Annual household income	<2.00	244	21.4	228	21.3	16	23.2	0.085
(million yen)	2.00–4.99	529	46.4	500	46.7	29	42	
	5.00–8.99	150	13.2	134	12.5	16	23.2	
	9.00+	48	4.2	46	4.3	2	2.9	
	Missing	169	14.8	163	15.2	6	8.7	
**Total score of recent life event stressors**		1097	57.5(61.1)	1032	56.7 −61	65	70.4 −60.2	0.079
**Current health status and social relationships**								
Total no of medical conditions (0–21)		1140	1.66 (1.23)	1071	1.64 (1.20)	69	199 (1.59)	0.022
Marital status	Not married	342	30	320	29.9	22	31.9	0.94
	Married	766	67.2	721	67.3	45	65.2	
	Missing	32	2.8	30	2.8	2	2.9	
Living arrangement	Alone	1016	89.1	954	89.1	62	89.9	0.84
	With someone	124	10.9	117	10.9	7	10.1	
Employment status	No	711	62.4	667	62.3	44	63.8	0.88
	Yes	325	28.5	307	28.7	18	26.1	
	Missing	104	9.1	97	9.1	7	10.1	
Giving support	No	271	23.8	256	23.9	15	21.7	0.68
	Yes	869	76.2	815	76.1	54	78.3	
Receiving support	No	300	26.3	284	26.5	16	23.2	0.54
	Yes	840	73.7	787	73.5	53	76.8	
Help-seeking attitude	Passive	435	38.2	411	38.4	24	34.8	0.62
	Neutral	386	33.9	358	33.4	28	40.6	
	Active	244	21.4	230	21.5	14	20.3	
	Missing	75	6.6	72	6.7	3	4.3	

**Table 2:** Prevalence ratio with 95%CI for association between Childhood Mental Health and Geriatric Depression after multiple imputation (*n* = 1140).

Variable	Crude		Model I		Model II		Model III		Model IV	
	PR (95%CI)		APR (95%CI)		APR (95%CI)		APR (95%CI)		APR (95%CI)	
Suicidal ideation (yes)	**1.58**	**(1.15–2.15)**	**1.55**	**(1.17–2.07)**	**1.40**	**(1.04–1.88)**	**1.38**	**(1.03–1.86)**	**1.38**	**(1.02–1.88)**

Note: PR = prevalence ration; APR = adjusted prevalence ratio; CI = confidence interval. Bold letters indicate statistically significant results where 95%CI did not include 1. Model I includes demographics and personality attributes; Model II includes Model I variables + childhood adversities; Model III includes Model II variables + adulthood adversities; Model IV includes Model III variables + current health status and social relations.

## Supplementary Materials

The following are available online at https://www.mdpi.com/1660-4601/17/7/2257/s1, Table S1: Medical conditions of the participants (*n* = 1140), Table S2: Prevalence ratio with 95% CI for association with Geriatric Depression after MI (*n* = 1140).
