# Supplementary material for: Association between Childhood Suicidal Ideation and Geriatric Depression in Japan: A Population-Based Cross-Sectional Study
Source: Int J Environ Res Public Health. 2020 Mar 27;17(7):2257. doi: 10.3390/ijerph17072257 (PMC7178046; doi:10.3390/ijerph17072257)
Supplement: Supplementary file 1 [file ijerph-17-02257-s001.pdf]

International Journal of
Environmental Research and Public Health

MDPI

Supplementary Materials

Supplementary Table 1. Medical conditions of the participants (n = 1,140).

**Variable**			**Suicidality in Childhood**				**p-Value**
	**All**		**No (*n* = 1071)**		**Yes (*n* = 69).**		
**Medical condition**	*n*	*n*	*n*	%	*n*	%	
Hypertension	620	54.4	588	54.9		46.4	0.17
Stroke	15	1.3	14	1.3	1	1.4	0.92
Heart diseases	116	10.2	101	9.4	15	21.7	0.001
Diabetes	172	15.1	160	14.9	12	17.4	0.58
Parkinson disease	5	0.4	5	0.5	0	0	0.57
Obesity (BMI≥30)	38	3.3	35	3.3	3	4.3	0.35
Psychopathology	9	0.8	8	0.7	1	1.4	0.52
Hyperlipidemia	189	16.6	173	16.2	16	23.2	0.13
Respiratory disease	48	4.2	45	4.2	3	4.3	0.95
Gastrointestinal disease	91	8	89	8.3	2	2.9	0.11
Urological disease	91	8	85	7.9	6	8.7	0.82
Musculoskeletal disease	110	9.6	100	9.3	10	14.5	0.16
Injury	15	1.3	12	1.1	3	4.3	0.02
Cancer	61	5.4	59	5.5	2	2.9	0.35
Hematological disease	9	0.8	7	0.7	2	2.9	0.04
Ophthalmological disease	201	17.6	181	16.9	20	29	0.01
Otorhinological disease	39	3.4	34	3.2	5	7.2	0.07
Sleep disorder	2	0.2	2	0.2	0	0	0.72
Other	97	8.5	90	8.4	7	10.1	0.62

Supplementary Table 2. Prevalence ratio with 95% CI for association with Geriatric Depression after MI (*n* = 1,140).

Variable.		Crude PR (95% CI)		Model I APR (95% CI)		Model II APR (95% CI)		Model III APR (95% CI)		Model IV APR (95% CI)	
Childhood mental health status											
Suicidal ideation (ref = No)	Yes	1.58	(1.15–2.15)	1.55	(1.17 – 2.07)	1.4	(1.04 – 1.88)	1.38	(1.03–1.86)	1.38	(1.02–1.88)
Basic demographics											
Age (years)		1.02	(1.01–1.03)	1.02	(1.01 – 1.03)	1.02	(1.01 – 1.03)	1.02	(1–1.03)	1.01	(0.99––1.02)
Sex (ref = Male)	Female	1.21	(0.99–1.47)	1.14	(0.94 – 1.38)	1.19	(0.98 – 1.45)	1.1	(0.9–1.35)	1.14	(0.91–1.43)
Personality attribute											
	Q2	1.49	(1.13–1.97)	1.29	(0.98 – 1.71)	1.29	(0.98 –1.7)	1.26	(0.95–1.66)	1.22	(0.93–1.6)
Neuroticism (ref = Q1)	Q3	2.01	(1.49–2.72)	1.61	(1.21 – 2.14)	1.57	(1.19 – 2.08)	1.5	(1.14–1.99)	1.39	(1.05–1.84)
	Q4	2.52	(1.93–3.29)	1.94	(1.48 – 2.53)	1.88	(1.44 – 2.45)	1.82	(1.39–2.37)	1.76	(1.36–2.28)
Extroversion (ref = Q1)	Q2	0.7	(0.55–0.89)	0.74	(0.58 – 0.94)	0.73	(0.58 – 0.93)	0.73	(0.58–0.93)	0.76	(0.59–0.96)
	Q3	0.56	(0.39–0.82)	0.66	(0.46 – 0.96)	0.65	(0.45 – 0.94)	0.67	(0.46–0.96)	0.67	(0.47–0.96)
	Q4	0.44	(0.33–0.59)	0.54	(0.4–0.73)	0.55	(0.41 – 0.74)	0.55	(0.41–0.75)	0.62	(0.46–0.83)
Agreeableness (ref = Q1)	Q2	0.65	(0.52–0.8)	0.68	(0.55 – 0.84)	0.67	(0.54 – 0.83)	0.66	(0.54–0.82)	0.68	(0.55–0.84)
	Q3	0.59	(0.41–0.84)	0.69	(0.48 – 0.97)	0.68	(0.48 – 0.96)	0.7	(0.49–0.98)	0.68	(0.49–0.95)
	Q4	0.29	(0.19–0.43)	0.41	(0.27 – 0.61)	0.41	(0.28 – 0.62)	0.42	(0.28–0.62)	0.43	(0.29 – 0.63)
Conscientiousness (ref = Q1)	Q2	0.74	(0.57–0.95)	0.77	(0.6–0.98)	0.79	(0.62 –1)	0.83	(0.65–1.06)	0.84	(0.66–1.06)
	Q3	0.73	(0.54–0.97)	0.71	(0.53 – 0.94)	0.72	(0.54 – 0.96)	0.78	(0.59–1.04)	0.81	(0.61–1.08)
	Q4	0.6	(0.43–0.84)	0.67	(0.48 – 0.93)	0.68	(0.48 – 0.95)	0.71	(0.51–0.99)	0.8	(0.57 – 1.11)

Openness (ref = Q1)	Q2	0.75	(0.56–1)	0.9	(0.67–1.21)	0.94	(0.7–1.26)	0.95	(0.71–1.27)	0.93	(0.7–1.24)
	Q3	0.66	(0.5–0.87)	0.81	(0.63–1.06)	0.83	(0.64–1.08)	0.82	(0.64–1.07)	0.85	(0.66–1.09)
	Q4	0.49	(0.34–0.69)	0.69	(0.48–0.97)	0.7	(0.49–0.99)	0.7	(0.5–0.99)	0.72	(0.51–1.01)
Childhood adversities											
ACE score (ref = 0)	1	1.67	(1.31–2.14)			1.52	(1.2–1.94)	1.45	(1.14–1.84)	1.44	(1.13–1.82)
	2+	1.83	(1.4–2.39)			1.53	(1.17–2.01)	1.45	(1.11–1.89)	1.35	(1.04–1.76)
Adulthood adversities											
Formal education (ref = <9 years)	10–12 years	0.82	(0.66–1.02)					1.02	(0.82–1.26)	1.05	(0.85–1.31)
	13 years +	0.6	(0.45–0.82)					0.86	(0.64–1.16)	0.91	(0.67–1.23)
Occupation (ref=manual/industrial laborer)	Skilled laborer	0.58	(0.41–0.84)					0.75	(0.52–1.07)	0.77	(0.53–1.1)
Annual household income (million yen) (ref = < 200)	200–500	0.6	(0.48–0.75)					0.74	(0.59–0.92)	0.79	(0.63–1)
	500–900	0.58	(0.41–0.81)					0.67	(0.47–0.94)	0.71	(0.49–1.01)
	900+	0.26	(0.10.64)					0.33	(0.14–0.8)	0.37	(0.15–0.91)
Total score of recent life event stressors		1	(1–1)					1	(1–1)	1	(1–1)
Current health status & social relationships											
Total number of medical conditions		1.13	(1.05–1.22)							1.07	(0.99–1.16)
Marital status (ref = Not married)	Married	0.65	(0.53–0.79)							0.83	(0.65–1.06)
Living arrangement (ref = Alone)	With someone	0.69	(0.53–0.9)							0.99	(0.73–1.35)
Employment status (ref = No)	Yes	0.7	(0.54–0.89)							0.83	(0.65–1.07)
Giving support (ref = No)	Yes	0.61	(0.5–0.75)							0.82	(0.63–1.06)
Receiving support (ref = No)	Yes	0.55	(0.46–0.67)							0.78	(0.61–1)
Help-seeking attitude (ref = Passive)	Neutral	0.52	(0.41–0.67)							0.63	(0.49–0.79)
	Active	0.59	(0.45–0.78)							0.78	(0.6–1.01)

Note: MI = multiple imputation; APR = adjusted prevalence ratio; CI = confidential interval. Bold letters indicate statistically significant results where 95%CI did not include 1. Model I includes childhood suicidal ideation, demographics and personality attributes; Model II includes Model I variables + childhood adversities; Model III includes Model II variables + adulthood adversities; Model IV includes Model III variables + current health status and social relations.
